# Alterations of ecosystem nitrogen status following agricultural land abandonment in the Karst Critical Zone Observatory (KCZO), Southwest China

**DOI:** 10.7717/peerj.14790

**Published:** 2023-01-27

**Authors:** Man Liu, Guilin Han

**Affiliations:** Institute of Earth Sciences, China University of Geosciences (Beijing), Beijing, China

**Keywords:** Soil N availability, NO_3_^–^ loss potential, 15N natural abundance, Soil aggregates, Secondary succession, Karst critical zone observatory

## Abstract

**Background:**

Secondary succession after agricultural land abandonment generally affects nitrogen (N) cycle processes and ecosystem N status. However, changes in soil N availability and NO_3_^–^ loss potential following secondary succession are not well understood in karst ecosystems.

**Methods:**

In the Karst Critical Zone Observatory (KCZO) of Southwest China, croplands, shrub-grass lands, and secondary forest lands were selected to represent the three stages of secondary succession after agricultural land abandonment by using a space-for-time substitution approach. The contents and ^15^N natural abundance (*δ*^15^N) of leaves, soils, and different-sized aggregates at the three stages of secondary succession were analyzed. The *δ*^15^N compositions of soil organic nitrogen (SON) in aggregates and soil to plant ^15^N enrichment factor (*EF* = *δ*^15^N_leaf_ −*δ*^15^N_soil_), combined with soil inorganic N contents and *δ*^15^N compositions were used to indicate the alterations of soil N availability and NO_3_^–^loss potential following secondary succession.

**Results:**

Leaf N content and SON content significantly increased following secondary succession, indicating N accumulation in the soil and plant. The *δ*^15^N values of SON also significantly decreased, mainly affected by plant *δ*^15^N composition and N mineralization. SON content in macro-aggregates and soil NH_4_^+^ content significantly increased while *δ*^15^N values of NH_4_^+^ decreased, implying increases in SON stabilization and improved soil N availability following secondary succession. Leaf *δ*^15^N values, the *EF* values, and the (NO_3_^–^-N)/(NH_4_^+^-N) ratio gradually decreased, indicating reduced NO_3_^–^ loss following secondary succession.

**Conclusions:**

Soil N availability improves and NO_3_^–^ leaching loss reduces following secondary succession after agricultural land abandonment in the KCZO.

## Introduction

Nitrogen (N) is a vital bioelement, which controls the primary productivity of many middle and high-latitude terrestrial ecosystems (Davidson et al., 2007; Vitousek & Howarth, 1991), and promotes plant and soil carbon (C) storage (Bae et al., 2015; Bell et al., 2020; Bell et al., 2021; Berg & Meentemeyer, 2002; Luo et al., 2004; Xia et al., 2021; Yang et al., 2016). Secondary succession after agricultural land abandonment, which is a common type of land-use change at the global scale, has been recognized to affect the ecosystem N cycle (Clark & Johnson, 2011; Djuma et al., 2020). Soil N processes, including N input, transfer, transform, and output processes, are the most important research contents of the ecosystem N cycle (Robinson, 2001). The natural abundance of stable N isotope (*δ*^15^N) has been widely used to indicate N sources and trace N processes (Boutton & Liao, 2010; Jiang et al., 2022; Kayler et al., 2011; Pardo et al., 2007). Input and transfer of soil N with discrepant *δ*^15^N values and ^15^N isotopic fractionation during soil N transformation and output processes change the *δ*^15^N composition of the original soil N pool (Currie, Nadelhoffer & Aber, 2004; Fowler et al., 2013; Liu et al., 2017; Martinez, Galantini & Duval, 2018; Waser et al., 1998; Zhang et al., 2015).

In agroecological systems, crops mainly absorb bioavailable N (NH_4_^+^ and NO_3_^−^) from chemical N fertilizer (Choi, Matushima & Ro, 2011; Muhammed et al., 2018). In addition, some leguminous crops can utilize atmospheric N through symbiotic N_2_ fixing (Hogberg, 1997). The ^15^N-abundance of synthetic fertilizer (*δ*^15^N: 0.3 ± 0.2‰) and atmospheric N_2_ (*δ*^15^N: 0‰) can significantly affect the *δ*^15^N composition of crops (Choi et al., 2017). However, after agricultural land abandonment, plants mainly absorb available N of the soil N pool by mineralization and nitrification (Hobbie & Ouimette, 2009). Soil N mineralization causes ^15^N-depletion in NH_4_^+^ and nitrification produces ^15^N-depletion in NO_3_^−^ (Baggs et al., 2003; Lim et al., 2015). Generally, soil available N is ^15^N-depleted and is easily absorbed or lost compared to soil organic nitrogen (SON) (Baggs et al., 2003; Corre et al., 2007). Denitrification produces ^15^N-depleted NOx and N_2_ that are released into the atmosphere (Robinson, 2001; Galloway et al., 2008). Ammonia volatilization causes ^15^N-depletion in gaseous NH_3_ and ^15^N-enrichment in residual NH_4_^+^ (Choi et al., 2017). As the ideal tracer of N sources and N processes, the ^15^N natural abundance of the soil likely has great potential in indicating the alterations of soil N processes following secondary succession after agricultural land abandonment.

The soil aggregate is the basic unit of soil structure and the main chamber of microbial activity (Blanco-Canqui & Lal, 2004). The formation and stabilization of aggregates are closely linked to the distributions of SON in different-sized aggregates (Ayoubi et al., 2012; Tripathi, Kushwaha & Singh, 2008). The response of SON in different-sized aggregates to land-use change is different, for example, there is a quicker response in macro-aggregates than in micro-aggregates (Lan, Hu & Fu, 2020). Thus, soil aggregates combined with ^15^N natural abundance can provide a clearer understanding of soil N dynamics under land-use change including secondary succession after agricultural land abandonment (Park et al., 2022).

The ^15^N natural abundance in soil-plant systems has been also widely employed to indicate ecosystem N status (Callesen et al., 2013; Garten et al., 2007; Pardo et al., 2007). Excess ^15^N-depleted NO_3_^−^ has to be lost in N-saturated ecosystems, which causes a ^15^N-enrichment in the soil N pool (Pardo et al., 2007). Increased soil *δ*^15^N value causes the ^15^N-enrichment in the plant because plant N is mainly derived from the soil N pool. The ^15^N-depleted available N is sufficiently absorbed by plants in N-limited ecosystems, resulting in foliar ^15^N depletion (Garten et al., 2007). The natural ^15^N-abundance of leaves has been widely used to indicate the N status of the forest ecosystem (Boeckx et al., 2005; Koopmans et al., 1997). However, the absolute value of foliar *δ*^15^N is unsuitable to compare the N statuses at different sites within a catchment, because foliar ^15^N-abundance is significantly affected by soil *δ*^15^N composition (Kahmen, Wanek & Buchmann, 2008; Liu, Yeh & Sheu, 2006; Ross, Lawrence & Fredriksen, 2004; Shan et al., 2019), which is generally discriminative at different sites (Taylor, Chazdon & Menge, 2019). Therefore, the *δ*^15^N value of surface soil is used to calculate the actual foliar ^15^N enrichment or depletion degree at a specific site, exhibited as the soil to plant ^15^N enrichment factor (*EF* = *δ*^15^N_leaf_ − *δ*^15^N_soil_, Pardo et al., 2007).

Generally, leaf *δ*^15^N values are lower than soil *δ*^15^N values, because the ^15^N-depleted available N is absorbed by plants (Baggs et al., 2003; Corre et al., 2007), thus the *EF* value is negative. The *EF* value is positively correlated with ecosystem N status (or the degree of NO_3_^−^ leaching) (Callesen et al., 2013). In closed N-cycling ecosystems, the ^15^N-depleted NO_3_^−^ is mainly absorbed by the plant, which causes ^15^N-depletion in the plant compared to soil (*i.e.,* the *EF* value is more negative). However, the *δ*^15^N values of the plants and soils are higher and the *EF* value is closer to zero with the long-term loss of the ^15^N-depleted NO_3_^−^ in N-saturated ecosystems (Callesen et al., 2013; Pardo et al., 2007). The application of the *EF* value has been mainly focused on the comparison of N status among different tree species and slope positions within a forest catchment (Callesen et al., 2013; Garten et al., 2007). However, a similar application by using the *EF* value in the indication of N status changes during secondary succession after agricultural land abandonment has not been reported. This study will be meaningful to expand the application of the *EF* value in ecosystems.

In the Karst Critical Zone Observatory (KCZO) of Southwest China, many croplands have been abandoned and naturally recovered with the promulgation of the Grain for Green Project (GGP) program in the 1990s (Wang et al., 2017). Changes in soil N content or stock following secondary succession after agricultural land abandonment have been widely reported in the karst ecosystem (Lan, Hu & Fu, 2020; Li et al., 2017; Wen et al., 2016; Xiao et al., 2018; Li et al., 2021) suggested that secondary succession significantly enhanced the gross N mineralization and nitrification rate, due to the increased gross microbial biomass and organic N input from plants. Although the increase in soil N availability enhances soil inorganic N supply, recoveries of plant biomass and microbe biomass (Li et al., 2018; Yang et al., 2017) likely can promote the uptake and assimilation of soil inorganic N. Thus, the changes in soil N processes following secondary succession are complex, which is associated with the interaction between soil, plant, and microbe. To identify the variation trends of soil N availability and soil N loss potential following secondary succession, we hypothesized that soil N availability enhances and NO_3_^−^ loss reduces after agricultural land abandonment in the KCZO. The ^15^N natural abundance in soil aggregates and the *EF* values are important research methods in this study. The study objectives were to: (1) identify the changes of *δ*^15^N compositions of SON in different-sized aggregate and the *EF* values at different stages following secondary succession and (2) estimate soil N availability and soil NO_3_^−^ loss potential following secondary succession after agricultural land abandonment in the KCZO.

## Material and Methods

### Study area

The study area is located in the Chenqi catchment (26°15.779′–26°16.710′N, 105°46.053 ′–105°46.839′E), with an area of 1.54 km^2^. The Chenqi catchment is one of the key research areas of the KCZO in Guizhou province, Southwest China. The climate in the catchment is mainly sub-tropical monsoonal, with a mean annual temperature (MAT) of 15.1 °C and mean annual precipitation (MAP) of 1,315 mm (Zhao et al., 2010). Seasonal evapotranspiration (mean: 260 mm in spring, 330 mm in summer, 185 mm in autumn, and 115 mm in winter) is much lower than seasonal precipitation (Gao et al., 2016). This catchment has a typical karst hoodoo depression landform, in which a valley is surrounded by three hills and a seasonal stream flows from east to west (Liu, Han & Zhang, 2020). The altitudes of these hills reach up to 1,524 m (above sea level) at their maximum, while the valley is only 1,310 m, with an average altitude of 1,350 m in this catchment (Yue et al., 2020). The soils on the hilltops and hillslopes are calcareous, mainly developed from the limestones of the upper and middle part of the Guanling Formation of the middle Triassic (Zhao et al., 2010), and classified as calcic Inceptisols in the soil taxonomy of the United States Department of Agriculture (USDA) (Soil Survey Staff, 2014). Quaternary deposits are mainly distributed on the valley floor, mainly deposited from the materials of surrounding hillslopes by soil erosion (Green, Dungait et al., 2019).

**Figure 1:**
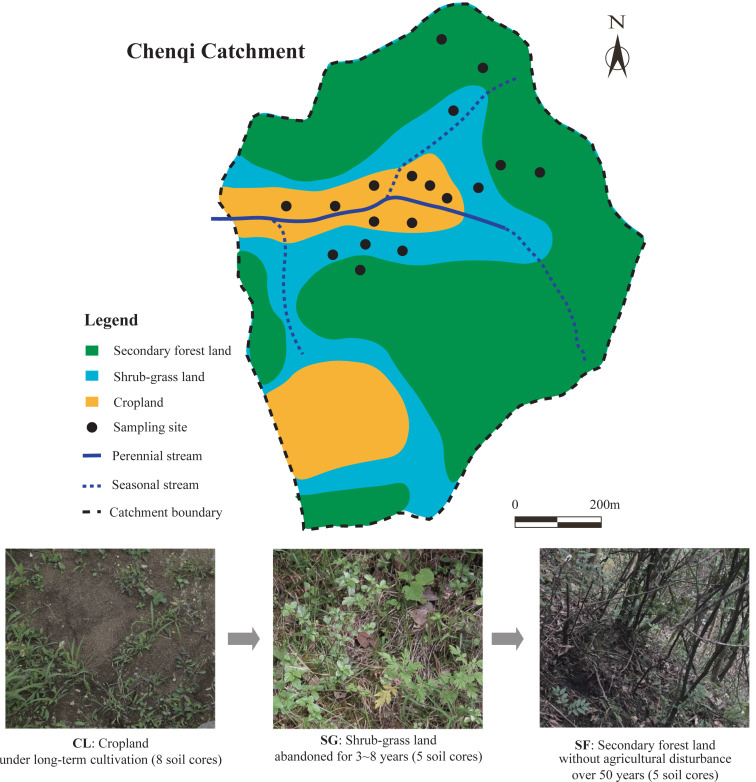
Map of sample sites in the Chenqi catchment and photographs of the three stages of secondary succession following agricultural land abandonment.

To restore the karst ecological environment, many sloping croplands with a low crop yield have been abandoned and naturally recovered under the GGP program since the 1990s (Wang et al., 2017). In the KCZO, a series of croplands were abandoned in different years, and gradually evolved into grassland, shrubland, and secondary forest lands in that order (Liu, Han & Zhang, 2020). The croplands (CL) on the valley floor remained in conventional cultivation or are in the fallow period, the shrub-grass lands (SG) at the foot of the hills have been transformed from terraced croplands for 3–8 years, and the secondary forest lands (SF) on the hillsides were converted from terraced croplands 50 years ago (Liu, Han & Zhang, 2020). Thus, the zone of croplands, shrub-grass lands, and secondary forest lands show a vertical distribution in the catchment. In the croplands, main crops such as maize (*Zea mays*), potato (*Solanum tuberosum*), oilseed rape (*Brassica napus*), and peanut (*Arachis hypogaea*) are planted in rotation from spring to autumn, while the fields lie fallow in winter (Qu & Han, 2022). N-P-K fertilizer and urea provide about 300 kg/ha N, 85 kg/ha P, and 6 kg/ha K per year for crop production, and farm manures are applied irregularly and non-quantitatively (Li et al., 2018). In the shrub-grass lands, the main plant species are herbaceous plants including *Imperata cylindrical*, *Setaria viridis*, and *Miscanthus sinensis*, and low shrubs or trees including *Berchemia sinica*, *Ilex macrocarpa*, and *Pyracantha fortuneana*. In the secondary forest lands, the main plant species are tall evergreen trees including *Litsea pungens*, *Padus racemosa*, *Pinus tabuliformis*, *Cinnamomum camphora*, *Camellia japonica*, and *Cyclocarya paliurus*. Above-ground biomass in the croplands, shrub-grass lands, and secondary forest lands are 2,020, 5,140, and 8,160 kg/ha, respectively; their below-ground biomass is 1,330, 2,740, and 1,570 kg/ha, respectively (Wang et al., 2021). The parent rock, soil type, and climate of different sites were similar in the small catchment. The average slope of the hills in the Chenqi catchment is more than 40° (Liu, Han & Zhang, 2020), which determines that the farmlands are designed as terraced croplands in order to avoid soil erosion. The topographic features of the different sites were similar, although there is a vertical distribution. In this study, the croplands, shrub-grass lands, and secondary forest lands are regarded as the different stages following secondary succession on abandoned croplands by the space-for-time substitution method (Blois et al., 2013). The photographs of the three stages following secondary succession are shown in Fig. 1.

### Sampling

Soil sampling was carried out in the Chenqi catchment in June 2016. At present, the proportional area of croplands, shrub-grass lands, and secondary forest lands within the Chenqi catchment is approximately 50%, 20%, and 30%, respectively. In total, 18 sampling sites from different land-use types were randomly selected related to the proportional area of each within the catchment, of which 8 sites were in the croplands, five sites were in the shrub-grass lands, and five sites were in the secondary forest lands. The location and land-use history of all sampling sites are shown in Table 1. The distance between two different sites under the same land-use type was 50 m to 100 m. A soil pit (0.5 m × 0.5 m × 0.5 m) was dug at each sample site, and three duplicate soil samples were chosen from the three sides of the pit. Soil samples were collected from the top to the bottom at 0–10, 10–20, and 20–30 cm depth. In total, 162 soil samples were collected. The duplicate samples in each pit at the same depth were mixed into one composite sample.

**Table 1:** Location and land-use history of sampling sites.

Site	longitude and latitude	Altitude (m)	Land-use history
Cropland			
CL1	26°15.797′N, 105°46.468′E	1,319	Maize land, long-term cultivation and fertilization
CL2	26°15.817′N, 105°46.267′E	1,320	Maize land, long-term cultivation and fertilization
CL3	26°15.806′N, 105°46.295′E	1,320	Peanut land, long-term cultivation and fertilization
CL4	26°16.010′N, 105°46.433′E	1,333	Vegetable garden mainly planted potato and scallions, long-term cultivation and fertilization
CL5	26°15.805′N, 105°46.278′E	1,334	Vegetable garden mainly planted sunflower, long-term cultivation and fertilization
CL6	26°16.010′N, 105°46.433′E	1,334	Maize land during the fallow period, without cultivation and fertilization for 1 year
CL7	26°15.872′N, 105°46.278′E	1,335	Maize land during the fallow period, without cultivation and fertilization for 2 years
CL8	26°16.019′N, 105°46.839′E	1,335	Maize land during the fallow period, without cultivation and fertilization for 2 years
Shrub-grass land			
SG1	26°15.893′N, 105°46.490′E	1,348	Without cultivation and fertilization for 3 years, covered by grasses
SG2	26°15.870′N, 105°46.495′E	1,350	Without cultivation and fertilization for 5 years, covered by shrub-grass
SG3	26°15.811′N, 105°46.291′E	1,365	Without cultivation and fertilization for 5 years, covered by shrub-grass
SG4	26°16.046′N, 105°46.543′E	1,370	Without cultivation and fertilization for 7 years, covered by shrub
SG5	26°16.021′N, 105°46.564′E	1,376	Without cultivation and fertilization for 8 years, covered by shrub
Secondary forest land			
SF1	26°16.091′N, 105°46.589′E	1,401	Without cultivation and fertilization >50 years, covered by secondary forest
SF2	26°15.984′N, 105°46.569′E	1,404	Without cultivation and fertilization >50 years, covered by secondary forest
SF3	26°15.980′N, 105°46.597′E	1,425	Without cultivation and fertilization >80 years, covered by secondary forest
SF4	26°15.779′N, 105°46.264′E	1,442	Without cultivation and fertilization >80 years, covered by secondary forest
SF5	26°16.125′N, 105°46.544′E	1,466	Without cultivation and fertilization >100 years, covered by secondary forest

The dominant vegetation species under the three land use types were identified in the field. Leaf samples from at least five plants with the same species were selected under the same land-use type. The mature leaves of the dominant vegetation species were collected at the high, middle, and low tree heights. The leaves from the same species were mixed to be one sample. In total, 37 leaf samples were collected, including nine in croplands, 13 in shrub-grass lands, and 15 in secondary forest lands.

### Sample analysis

After washing off the dust on the leaf surface with pure water 3 times, the leaf samples were dried at −40 °C in a freezer dryer, then ground into powder by an attritor. Soil samples were air-dried (25 °C) after removing obvious gravel and fresh coarse roots. A part of the samples was crushed by hand to make all particles pass through a 10 mesh-steel sifter (2 mm), which was stored as the sample of bulk soil (<2 mm). The remaining soil samples were not crushed and were used for soil aggregate separation by using the improved wet-sieving method (Six et al., 2002). Macro-aggregates (250–2000 µm), micro-aggregates (53–250 µm), and silt + clay sized fractions (<53 µm) were collected after passing through 250 µm and 53 µm sifters, respectively. The moist aggregate samples were dried in an oven at 55 °C until constant weight and then weighed to calculate their mass percent.

The samples of bulk soils and different-sized aggregates were ground in an agate mortar until all fine particles pass through a 200 mesh-nylon sifter. The powder samples (<75 µm) were soaked in a 2 mol/L KCl solution for 24 h to remove NO_3_^−^, NH_4_^+^, and dissolved organic N (Meng, Ding & Cai, 2005). The treated samples were washed repeatedly with pure water until all Cl^−^ was removed, then dried and ground into powder for analyses of N content and *δ*^15^N composition.

The foliar N contents and SON contents of bulk soils and aggregates were measured by a multi-elemental analyzer (Vario TOC cube; Elementar, Hessen, Germany) in the Surficial Environment and Hydrological Geochemistry Laboratory, China University of Geosciences (Beijing, China). Standard soil substances (OAS B2152) were repeatedly measured to monitor reproducibility. The relative standard deviations were less than 3%. Actual SON content could be calibrated because of sample mass reduction after removing dissolved inorganic and organic N, *i.e.,* the measured SON content is multiplied by the ratio of the sample mass after and before treatment (Liu, Han & Li, 2021).

The N stable isotope ratio (^15^N/^14^N) of leaf, SON in bulk soils and aggregates were determined by an isotope mass spectrometer (MAT-253, Thermo, USA) in the Central Laboratory for Physical and Chemical Analysis, Institute of Geographic Sciences and Natural Resources Research, Chinese Academy of Sciences. The measurements are expressed in standard *δ* notation (‰) to indicate the differences between the ^15^N/^14^N ratio of the samples and accepted standard materials (atmospheric N_2_), where (Han et al., 2020):

(1) \begin{eqnarray*}{\delta }^{15}{N}_{\mathrm{ sample}}(\permil )=(({R}_{\mathrm{sample}}-{R}_{\mathrm{air}})/{R}_{\mathrm{air}})\times 1000,R={}^{15}N{/}^{14}N.\end{eqnarray*}

Reference material (GBW04494, *δ*^15^N_Air_: 0.24‰ ± 0.13‰) was monitored and repeatedly measured to evaluate the precision of the measurements (<0.1‰).

### Statistical analysis

One-way ANOVA with the least significant difference (LSD) test was conducted to identify the significant differences in SON contents of aggregates, *δ*^15^N values of leaves and SON in bulk soils and aggregates, and the *EF* values among the three land-use types at the significance level of *P* < 0.05. Linear and non-linear regression analyses were used to determine the variations of foliar N contents and C/N ratios following secondary succession on abandoned croplands. Statistical analyses were performed using the SPSS 18.0 (SPSS Inc., Chicago, IL, USA) software program and the figures were drawn using SigmaPlot 12.5 (Systat Software GmbH, Erkrath, Germany) software program.

## Results

### Foliar N contents and C/N ratios

**Figure 2:**
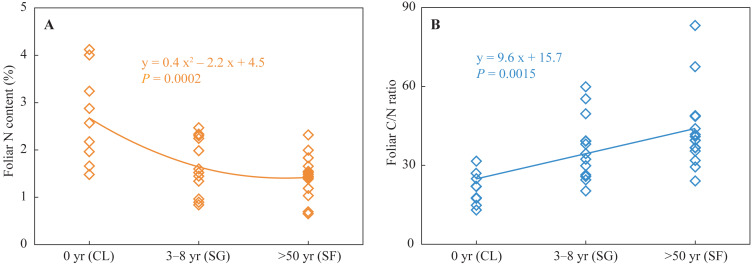
Foliar N content and C/N ratio at three stages of secondary succession following agricultural land abandonment. CL, cropland; SG, shrub-grass land; and SF, secondary forest land.

Foliar N contents in the croplands were higher than those in the shrub-grass lands and much higher than those in the secondary forest lands (Fig. 2). Foliar C/N ratios in the croplands were lower than those in the shrub-grass lands and much lower than those in the secondary forest lands (Fig. 2). Foliar N contents decreased while C/N ratios increased following secondary succession on abandoned croplands.

### SON contents of bulk soils and aggregates

**Figure 3:**
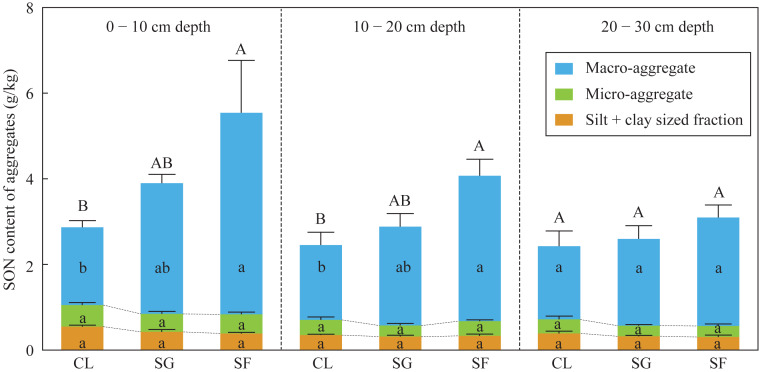
The SON content of bulk soils and different-sized aggregates at three stages of secondary succession following agricultural land abandonment. Error bar is standard error (SE). SON content of bulk soils is equal to the summation of SON contents of macro-aggerates, macro-aggerates, and silt + clay sized fractions. Different uppercase letters indicate significant differences in SON contents of bulk soils among different stages at the same soil depth, different lowercase letters indicate significant differences in SON contents of macro-aggerates, macro-aggerates, or silt + clay sized fractions among different stages at the same soil depth, based on the one-way ANOVA with LSD test at the level of *P* < 0.05. CL, cropland; SG, shrub-grass land; and SF, secondary forest land.

The SON contents of bulk soils and macro-aggregates at the 0–20 cm depth in the croplands were slightly lower than those in the shrub-grass lands, and significantly lower than those in the secondary forest lands (Fig. 3). While the SON contents of macro-aggregates and silt + clay sized fractions at the 0–20 cm depth were not significantly different among the three land-use types (Fig. 3). Moreover, in the soils at the 0–20 cm depth, the SON contents of bulk soils, macro-aggregates, micro-aggregates, and silt + clay sized fractions were not significantly different among the three land-use types (Fig. 3). SON contents of bulk soils and macro-aggregates in the surface soils (0–20 cm depth) significantly increased following secondary succession on abandoned croplands.

### Foliar *δ*^15^N values and soil to plant ^15^N *EF* values

**Figure 4:**
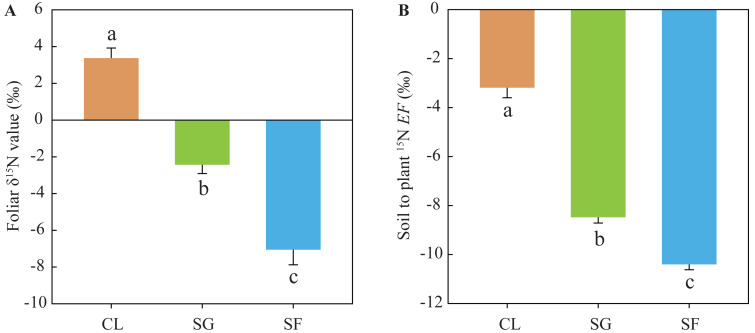
(A) Foliar *δ*^15^N values and (B) soil to plant ^15^*EF* values at three stages of secondary succession following agricultural land abandonment. Error bar is standard error (SE). *EF* = *δ*^15^N_leaf_ − *δ* ^15^N_soil_, *δ*^15^N_leaf_ is the foliar *δ*^15^N value, *δ*^15^N_soil_ is the *δ*^15^N value of SON in bulk soil at the 0–10 cm depth. Different lowercase letters indicate significant differences in foliar *δ*^15^N values or *EF* values among different stages, based on the one-way ANOVA with LSD test at the level of *P* < 0.05. CL, cropland; SG, shrub-grass land; and SF, secondary forest land.

Foliar *δ*^15^N values in the croplands (mean 3.37‰) were significantly higher than those in the shrub-grass lands (mean −2.43‰), and much higher than those in the secondary forest lands (mean −7.06‰) (Fig. 4). Similarly, the soil to plant ^15^N *EF* values in the croplands (mean −3.19‰) were significantly higher than those in the shrub-grass lands (mean −8.48‰), and much higher than those in the secondary forest lands (mean –10.40‰) (Fig. 4). Foliar *δ*^15^N values and the *EF* values significantly decreased following secondary succession on abandoned croplands.

### *δ*^15^N values of SON in bulk soils and aggregates

**Figure 5:**
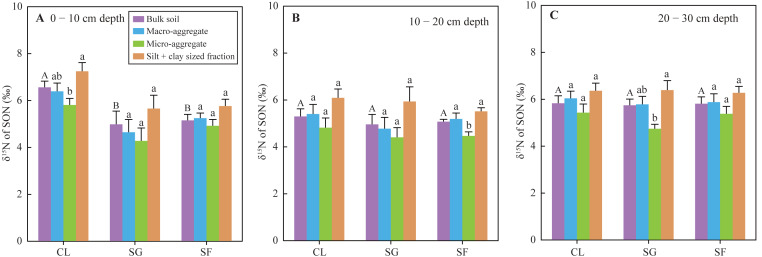
(A–C) The *δ*^15^N values of SON in bulk soils and different-sized aggregates at three stages of secondary succession following agricultural land abandonment. Error bar is standard error (SE). Different uppercase letters indicate significant differences in *δ*^15^N values of SON in the bulk soils at the same depth among different stages; different lowercase letters indicate significant differences in soil *δ*^15^N values of SON among different-sized aggregates at the same stage and depth, based on the one-way ANOVA with LSD test at the level of *P* < 0.05. CL, cropland; SG, shrub-grass land; and SF, secondary forest land.

The *δ*^15^N values of SON in bulk soils at the 0 −10 cm depth under the croplands (mean 6.56‰) were significantly higher than those under the shrub-grass lands (mean 4.99‰) and secondary forest lands (mean 5.16‰), while the *δ*^15^N values in the soil of the 10–30 cm depth were not significantly different among the three land-use types (Fig. 5). The SON of top soils (0–10 cm depth) gradually enriched ^14^N following secondary succession on abandoned croplands. Additionally, the *δ*^15^N values of SON in different sized aggregate at all soil depths and under all land-use types varied following the order: micro-aggregates <macro-aggregates <silt + clay sized fractions (Fig. 5). Although these differences of *δ*^15^N values between different sized aggregates were not always significant statistically, the same feature of aggregate *δ*^15^N-discrepancies in all soil samples.

## Discussion

### Improved soil N availability following secondary succession

Soil N availability reflects the bioavailable N supply capacity of the soil, which is mainly affected by SON contents and the reaction rate of N mineralization (Tang et al., 2017). SON is the main substrate derived from plant N and biological N fixation (Vitousek et al., 2013). In the croplands, extraneous N fertilizer is absorbed by crops to enhance its N content, thus foliar N contents were higher than those in the shrub-grass lands and secondary forest lands (Fig. 2). Although foliar N contents decreased after agricultural land abandonment, increased plant biomass enhance N input by litters and root exudates (Wang et al., 2021). Besides N inputs from plants, biological N fixation also make a considerable contribution to soil N inputs (Vitousek et al., 2013). Li et al. (2018) found the absolute abundances of N functional genes for N_2_ fixation gradually increased following secondary succession in the study area, indicating enhanced soil N stock by biological N fixation. Thus, increases in N input from plant and biological N fixation promote SON accumulation in the surface soils after agricultural land abandonment (Fig. 3).

Soil NH_4_^+^-N contents at the 0–30 cm depth significantly increased following secondary succession (Table S2), which suggests an accelerated N mineralization rate. The observed soil NH_4_^+^-N content is not the gross yield of N mineralization, which is the remainder after deducting absorption by plant and soil microbes, as well as NH_4_^+^ leaching loss (despite it being negligible) (Galloway et al., 2008; Robinson, 2001). However, absorbed NH_4_^+^ by plant and soil microbes also increase due to increases in plant biomass and microbial biomass (Guo et al., 2021; Wang et al., 2021; Zhang et al., 2000). Thus, it is likely that the soil N mineralization rate is rapid after agricultural land abandonment. This is also supported by the changes in *δ*^15^N composition of SON and foliar N following secondary succession. Mean foliar *δ*^15^N values in the croplands, shrub-grass lands, and secondary forest lands were 3.37‰, −2.43‰, and −7.06‰, respectively (Fig. 4), while the *δ*^15^N values of SON in top soils (0–10 cm depth) under the three land-use types was 6.56‰, 4.99‰, and 5.16‰, respectively (Fig. 5). The *δ*^15^N composition of SON in top soils is influenced by the natural ^15^N-abundance of the source plants (Boeckx et al., 2005), and is also affected by *δ*^15^N fractionation during N mineralization (Baggs et al., 2003; Corre et al., 2007). Soil N mineralization leads to ^15^N enrichment in residual SON and produces ^15^N-depleted NH_4_^+^ (Baggs et al., 2003; Corre et al., 2007). SON in top soils enriches more ^15^N compared to litter (or foliar) N following secondary succession, indicating an intensive N mineralization process. In summary, increases in N input from plants, biological N fixation, and soil N mineralization rate determine soil N availability during secondary succession.

### Reduced soil NO_3_^−^ loss following secondary succession

Foliar *δ*^15^N values and soil to plant ^15^N *EF* values significantly decreased following secondary succession (Fig. 4), indicating reduced soil N losses after agricultural land abandonment (Pardo et al., 2007). Wang et al. (2021) found denitrification was unlikely to be a major contribution to soil NO_3_^−^ loss, based on the weak correlation between *δ*^15^N and *δ*^18^O of NO_3_^−^ in this study area. Thus, soil NO_3_^−^ leaching is the main cause of losses in this catchment. Furthermore, the (NO_3_^−^-N)/(NH_4_^+^-N) ratio gradually decreased following secondary succession in the study area (Table S1), suggesting a reduced risk of soil NO_3_^−^ leaching (Wang et al., 2021). Soil NO_3_^−^ loss potential decreased following secondary succession, mainly attributed to increased SON stabilization and soil inorganic N uptake capacity.

Foliar N contents decreased, while C/N ratios increased following secondary succession (Fig. 2), which could cause similar variations in the N contents and C/N ratios of litter. Generally, the decomposition of litter with a low N concentration and high C/N ratio will be restrained at the initial stage of litter decomposition (Xia et al., 2021). Thus, the fresh SON derived from litter in the shrub-grass lands and secondary forest lands is more stable than that in the croplands. SON contents of bulk soils and macro-aggregates in the surface soils significantly increased following secondary succession, but SON contents of micro-aggregates and silt + clay sized fractions did not significantly vary (Fig. 3). Moreover, macro-aggregate proportions and the contribution of macro-aggregates to SON stock in the surface soils also significantly increased following secondary succession (Table S1). These results indicate that added SON after agricultural land abandonment is mainly stored within macro-aggregates. Physical protection of macro-aggregates by isolating oxygen, water, microbes, and enzymes also enhances SON stabilization (Six et al., 2002; Wright & Hons, 2005; Zhu, Deng & Shangguan, 2018). In addition, the *δ*^15^N values of SON in micro-aggregates were always lower than that in macro-aggregates (Fig. 5), which was closely linked to the different degrees of N mineralization. Due to ^15^N enrichment in residual SON during the N mineralization process (Baggs et al., 2003; Corre et al., 2007), it can be inferred that the SON in micro-aggregates is fresher than that in macro-aggregates, which is consistent with the findings based on *δ*^13^C values of SOC (Liu, Han & Zhang, 2020). According to the aggregate hierarchy model (Six, Elliott & Paustian, 2000), micro-aggregates are formed within macro-aggregates, resulting in more fresh-SON combined within micro-aggregates. The SON within micro-aggregates is more stable because of the multiple protections of micro-aggregates and macro-aggregates (Beare, Hendrix & Coleman, 1994). The stability of SON is improved after agricultural land abandonment due to the increased C/N ratio of litter and aggregate protection. During secondary succession, the total N mineralization rate is improved, as reflected by the increased NH_4_^+^ contents (Table S2). The ratio of mineralized SON in total SON is likely reduced due to increased SON stability, which may restrict N mineralization and subsequent nitrification to produce NO_3_^−^ in unit time and unit SON. Thus, improved SON stability is meaningful to reduce soil NO_3_^−^ loss potential following secondary succession.

Soil NO_3_^−^ production and consumption (mainly including uptake by plants and microbes, leaching loss, and residual NO_3_^−^ in soils) at all stages of secondary succession must be balanced (Dai et al., 2020; Soper et al., 2018). Nitrification causes ^15^N enrichment in residual NH_4_^+^ and produces ^15^N-depleted NO_3_^−^ (Lim et al., 2015). The *δ*^15^N values of NH_4_^+^ increased and the *δ*^15^N values of NO_3_^−^ decreased following secondary succession (Table S2), indicating an intensive nitrification process and increased NO_3_^−^ production after agricultural land abandonment (Wang et al., 2021). Soil NO_3_^−^ leaching loss and residual soil NO_3_^−^ (Table S2) gradually decreased following secondary succession. Thus, it is likely that soil NO_3_^−^ uptake by plants and microbes is enhanced after agricultural land abandonment, which is mainly attributed to increases in plant biomass and microbial biomass. N is a bioelement; the growth and reproduction processes of plant and soil microbes need to assimilate bioavailable N (including NO_3_^−^-N) from soils (Zhang et al., 2000). In the study area, plant biomass and soil microbial necromass increased following secondary succession (Guo et al., 2021; Wang et al., 2021), increasing demands for bioavailable N.

**Figure 6:**
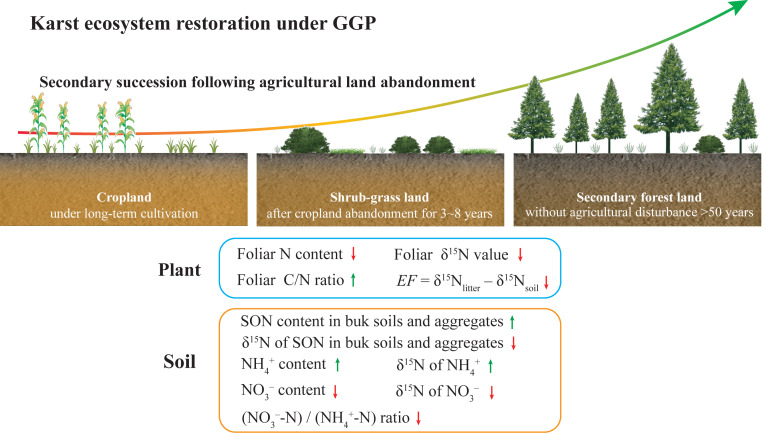
A diagram for changes in plant and soil N content and *δ*^15^N values during secondary succession after agricultural land abandonment under GGP. The green arrows indicate increase; the red arrows indicate decrease. The data of NH_4_^+^ and NO_3_^–^ content, (NO_3_^–^-N)/(NH_4_^+^-N) ratio, and *δ*^15^N value of NH_4_^+^ and NO_3_^–^ were reported by Wang et al. (2021).

In the present study, the diagram exhibits the changes in plant and soil N content and *δ*^15^N values during secondary succession after agricultural land abandonment under GGP as shown in Fig. 6, which reflects improved soil N availability and reduced soil NO_3_^−^ loss following secondary succession. Our study highlights the positive implications of the GGP program for karst ecosystem restoration. As the GGP program in China progresses, this conceptual model can provide basic scientific guidance for policymakers in the karst region, and even in the non-karst region. However, considering the differences in climate, soil type, and vegetation restoration approach in different regions, the key factor that affected soil N transformation processes and N loss may differ (Osborne et al., 2017). For future research, the conceptual model should be improved to make it applicable in other environments, particularly non-karst.

## Conclusions

Under the GGP program, the changes in soil N availability and soil NO_3_^−^ loss potential following secondary succession after agricultural land abandonment were estimated in the KCZO, Southwest China. During secondary succession, soil N input from plant and biological N fixation promote SON accumulation in the surface soils. Increases in soil SON stock and N mineralization rate determine soil N availability. Decreases in foliar *δ*^15^N values and soil to plant ^15^N *EF* values indicate reduced soil N losses. Improved SON stability by increasing the C/N ratio of litter and aggregate protection and increased soil NO_3_^−^ uptake by plants and microbes are beneficial for reducing soil NO_3_^−^ loss potential. This study highlights the positive implications of the GGP program for karst ecosystem restoration. In future works, the evaluation of soil N availability and the mechanism of soil NO_3_^−^ loss potential following secondary succession after agricultural land abandonment should be studied in other environments, particularly non-karst.

##  Supplemental Information

10.7717/peerj.14790/supp-1 Supplemental Information 1 Supplementary tables

10.7717/peerj.14790/supp-2 Supplemental Information 2 Raw data

## Abbreviations

*δ*^15^N: Stable nitrogen isotopic composition
ANOVA: Analysis of Variance
CL: Cropland
EF: Enrichment Factor
GGP: Grain for Green Project
KCl: Potassium chloride
KCZO: Karst Critical Zone Observatory
LSD: Least significant difference
MAP: Mean annual precipitation
MAT: Mean annual temperature
N, N_2_: Nitrogen
NH_3_: Ammonia
NH_4_^+^: Ammonium
NO_3_^−^: Nitrate
NO_x_: Nitrogen oxides
N_2_O: Nitrous oxide
SF: Secondary forest land
SG: Shrub-grass lands
SOC: Soil organic carbon
SON: Soil organic nitrogen
USDA: United States Department of Agriculture

## Additional Information and Declarations

### Data Availability

The following information was supplied regarding data availability: The raw measurements are available in the Supplementary File.
