# Supplementary material for: Alterations of ecosystem nitrogen status following agricultural land abandonment in the Karst Critical Zone Observatory (KCZO), Southwest China
Source: PeerJ. 2023 Jan 27;11:e14790. doi: 10.7717/peerj.14790 (PMC9885863; doi:10.7717/peerj.14790)
Supplement: Supplemental Information 1 [file peerj-11-14790-s001.docx]

The contribution G*_k_* (%) of different-sized aggregates to SON stock was calculated, as follows:

$$\text{G}_{\text{k}}\text{ = }\frac{\text{N}_{\text{k}}\text{ ×}\text{ M}_{\text{k}}}{\sum_{\text{k}\text{=1}}^{\text{n}} \text{(N}_{\text{k}}\text{ ×}\text{ M}_{\text{k}}\text{)}}\text{ ×100\%}$$

where *k* is aggregate class (*k* = 1, 2, 3 indicates macro-aggregate, microaggregate, and silt + clay sized fraction, respectively); N*_k_* (g/kg) is the SON content of the sized aggregate; and M*_k_* (%) is the mass proportion of the sized aggregate.

**Table S1** Proportion of different sized aggregates and the contribution of them to SON stock under different land-use types.

|  | Soil at the 0–10 cm depth | | |  | Soil at the 10–20 cm depth | | |  | Soil at the 20–30 cm depth | | |
| --- | --- | --- | --- | --- | --- | --- | --- | --- | --- | --- | --- |
|  | Cropland | Shrub-grass land | Secondary forest land |  | Cropland | Shrub-grass land | Secondary forest land |  | Cropland | Shrub-grass land | Secondary forest land |
| Proportion of macro-aggregates (%) | 61.3±5.8 b | 78.5±3.7 a | 81.6±5.9 a |  | 66.3±14.8 b | 79.3±3.1 ab | 82.6±3.9 a |  | 64.0±18.6 a | 76.0±3.7 a | 81.2±5.9 a |
| Proportion of micro-aggregates (%) | 15.8±2.1 a | 9.2±1.8 b | 8.1±1.6 b |  | 15.0±5.9 a | 8.5±2.3 ab | 7.7±1.4 b |  | 14.1±5.1 a | 9.8±1.0 ab | 7.4±1.7 b |
| Proportion of silt + clay sized fractions (%) | 22.9±5.7 a | 12.3±2.2 b | 10.3±4.4 b |  | 18.8±10.0 a | 12.2±1.6 a | 9.7±3.1 a |  | 21.9±15.70 a | 14.2±2.9 a | 11.4±5.2 a |
| Contribution of macro-aggregates to SON stock (%) | 64.4±3.8 b | 78.5±3.1 a | 82.9±4.8 a |  | 68.0±12.5 b | 79.8±3.5 ab | 83.1±4.1 a |  | 64.9±17.5 a | 77.0±4.3 a | 81.7±5.2 a |
| Contribution of micro-aggregates to SON stock (%) | 17.3±2.5 a | 11.4±1.7 b | 9.3±2.0 b |  | 15.5±5.7 a | 9.5±2.5 ab | 8.8±1.8 b |  | 15.1±5.7 a | 10.4±1.5 a | 8.7±2.0 a |
| Contribution of silt + clay sized fractions to SON stock (%) | 18.3±3.4 a | 10.0±1.9 b | 7.9±2.9 b |  | 16.5±8.0 a | 10.7±1.7 a | 8.0±2.7 a |  | 20.0±13.1 a | 12.6±3.0 a | 9.6±4.0 a |

The result expresses as mean and standard deviation. Different lowercase letters indicate significant differences in aggregate proportion or contribution to SON stock among different land-use types at the same soil depth, based on the one-way ANOVA with LSD test at the level of *P* < 0.05.

**Table S2** Soil inorganic N content and their δ^15^N value under different land-use types.

|  | Soil at the 0–10 cm depth | | |  | Soil at the 10–20 cm depth | | |  | Soil at the 20–30 cm depth | | |
| --- | --- | --- | --- | --- | --- | --- | --- | --- | --- | --- | --- |
|  | Cropland | Shrub-grass land | Secondary forest land |  | Cropland | Shrub-grass land | Secondary forest land |  | Cropland | Shrub-grass land | Secondary forest land |
| NH_4_^+^ content (mg/kg) | 4.29±0.15 b | 6.72±0.08 b | 24.10±2.25 a |  | 7.08±1.54 b | 7.13±0.37 b | 15.51±1.15 a |  | 4.36±0.27 b | 9.82±2.05 a | 9.57±0.38 a |
| NO_3_^–^ content (mg/kg) | 21.93±3.48 a | 11.84±1.95 b | 0.93±0.13 c |  | 22.22±4.90 a | 7.73±0.22 b | 0.80±0.49 c |  | 13.23±0.93 a | 1.40±0.76 b | 0.95±0.01 b |
| (NO_3_^–^-N)/(NH_4_^+^-N) ratio | 5.06±0.61 a | 1.77±0.31 b | 0.04±0.01 c |  | 3.49±0.96 a | 1.09±0.09 b | 0.06±0.04 c |  | 3.03±0.06 a | 0.12±0.05 b | 0.11±0.00 b |
| δ^15^N of NH_4_^+^ (‰) | -3.50±2.36 b | -1.49±0.68 b | 1.56±0.70 a |  | -3.41±3.27 a | -2.75±1.48 a | 1.55±2.51 a |  | -5.05±1.15 a | -3.38±1.78 a | 0.66±2.88 a |
| δ^15^N of NO_3_^–^ (‰) | 4.70±2.25 a | -8.26±5.59b | -13.88±3.52 b |  | 4.42±2.32 a | -9.43±3.62 b | -13.20±3.56 b |  | 3.60±1.26 a | -10.74±1.63 b | -9.38±6.61 b |

The result expresses as mean and standard deviation. Different lowercase letters indicate significant differences in soil inorganic N content and their δ^15^N value among different land-use types at the same soil depth, based on the one-way ANOVA with LSD test at the level of *P* < 0.05. All data are referred to *Wang et al., 2021*.
